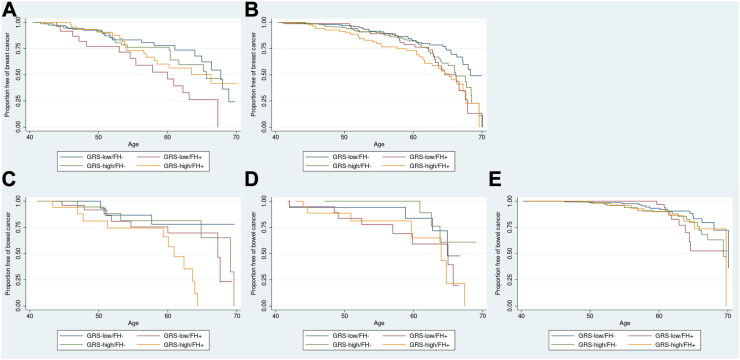# Influence of family history on penetrance of hereditary cancers in a population setting

**DOI:** 10.1016/j.eclinm.2023.102159

**Published:** 2023-09-14

**Authors:** Leigh Jackson, Michael N. Weedon, Harry D. Green, Bethan Mallabar-Rimmer, Jamie W. Harrison, Andy R. Wood, Kate S. Ruth, Jess Tyrrell, Caroline F. Wright

**Affiliations:** Institute of Biomedical and Clinical Science, University of Exeter College of Medicine and Health, RILD Building, Royal Devon & Exeter Hospital, Barrack Road, Exeter, EX2 5DW, UK

**Keywords:** Penetrance, Inheritance, Family history, BRCA, Lynch syndrome

## Abstract

**Background:**

We sought to investigate how penetrance of familial cancer syndromes varies with family history using a population-based cohort.

**Methods:**

We analysed 454,712 UK Biobank participants with exome sequence and clinical data (data collected between March 2006 and June 2021). We identified participants with a self-reported family history of breast or colorectal cancer and a pathogenic/likely pathogenic variant in the major genes responsible for hereditary breast cancer or Lynch syndrome. We calculated survival to cancer diagnosis (controlled for sex, death, recruitment centre, screening and prophylactic surgery).

**Findings:**

Women with a pathogenic *BRCA1* or *BRCA2* variant had an increased risk of breast cancer that was higher in those with a first-degree family history (relative hazard 10.3 and 7.8, respectively) than those without (7.2 and 4.7). Penetrance to age 60 was also higher in those with a family history (44.7%, CI 32.2–59.3 and 24.1%, CI 17.5–32.6) versus those without (22.8%, CI 15.9–32.0 and 17.9%, CI 13.8–23.0). A similar pattern was seen in Lynch syndrome: individuals with a pathogenic *MLH1*, *MSH2* or *MSH6* variant had an increased risk of colorectal cancer that was significantly higher in those with a family history (relative hazard 35.6, 48.0 and 9.9) than those without (13.0, 15.4 and 7.2). Penetrance to age 60 was also higher for carriers of a pathogenic *MLH1* or *MSH2* variant in those with a family history (30.9%, CI 18.1–49.3 and 38.3%, CI 21.5–61.8) versus those without (20.5% CI 9.6–40.5 and 8.3% CI 2.1–30.4), but not for MSH6 (6.5% CI 2.7–15.1 with family history versus 8.3%, CI 5.1–13.2). Relative risk increases were also observed both within and across conditions.

**Interpretation:**

Individuals with pathogenic cancer syndrome variants may be at a less elevated risk of cancer in the absence of a first-degree family history, so in the context of results return, family history should be considered when counselling patients on the risks and benefits of potential follow-up care.

**Funding:**

The current work is supported by the 10.13039/501100000265MRC (grant no MR/T00200X/1). The MRC had no role in the design and conduct of the study; collection, management, analysis, and interpretation of the data; preparation, review, or approval of the manuscript; and decision to submit the manuscript for publication.

**Research in context**

**Evidence before this study**

At the initiation of the study (1st June 2021), we searched the PubMed database using the search terms [(BRCA1 OR BRCA2 OR MLH1 OR MSH2 OR MSH6) AND (family history OR familial OR penetrance)] and identified a number of relevant studies. The literature concerning pathogenic variants for cancer predisposition in these studies is currently focussed around penetrance estimates in clinically ascertained cohorts and the effects of family history on this. Where population cohort data does exist, family history information is often not available or not interrogated to inform penetrance estimates.

**Added value of this study**

This study shows for the first time, using data from a large population cohort, that much of the risk conferred by a rare pathogenic variant associated with hereditary breast and ovarian cancer or Lynch syndrome is conferred by a first-degree family history of disease. This difference in penetrance in carrier individuals could be sufficient to impact on whether an individual might be eligible for specialist clinical care (e.g., National Institute for Health and Care Excellence (NICE) guidance on familial breast cancer requires ≥30% lifetime risk to be eligible for referral to a specialist genetic clinic in the UK).

**Implications of all the available evidence**

It is imperative that individuals who receive genotype information indicating a predisposition to cancer are appropriately counselled as to their individual risk profile in the context of their family history of disease. For those ascertained outside of the standard clinical pathway, this will help to provide patients with more accurate information to allow them to make informed decisions about prophylactic options.

## Introduction

Genetic testing for inherited cancer syndromes is offered to affected individuals based on various qualifying criteria.^1^^,^^2^ For example, *BRCA1* and *BRCA2* testing is offered to individuals with breast or ovarian cancer and/or a known history of hereditary breast and ovarian cancer (HBOC)^3^ in many countries, including the UK and USA, although there have been suggestions that this should be expanded to all women diagnosed with breast cancer.^4^ Similarly, patients presenting with colorectal cancer are routinely offered screening for hereditary non-polyposis colorectal cancer (HNPCC, or Lynch syndrome).^5^ If a causal pathogenic variant is identified, chemoprevention or prophylactic mastectomy/oophorectomy may be advised for HBOC, or regular colonoscopies or prophylactic surgery for Lynch syndrome.^6^^,^^7^ However, if no causal variant is found, individuals with a family history of breast or colorectal cancer still have a higher cancer risk relative to the wider population, indicating as yet unknown variants, genes or further risk factors are involved.^8^^,^^9^

Traditionally, clinical genetics has used a phenotype-first approach to identify the most likely cases with an underlying genetic pathology.^10^ Some pathogenic variants in genetic conditions have incomplete penetrance (where only a proportion of variant carriers will develop the condition), leading to reduced risk of disease.^11^ In the context of hereditary cancer syndromes, by offering genetic testing only to those individuals who have a family history of cancer, there is an inherent ascertainment bias towards finding highly penetrant variants in those families; the variant must have segregated through the family and been detected in an individual with cancer. This bias can lead to artificially high estimates of the penetrance of some variants. However, if found incidentally in an individual with no family history of disease, the penetrance of the same pathogenic variant is unlikely to be as high.^12^

Increasingly, a genotype-first approach is being used to identify individuals with pathogenic variants.^13^, ^14^, ^15^ The challenges of reporting and interpreting variants discovered in the absence of a phenotype have been explored.^16^ Population sampling of unselected individuals is required for calculating prevalence and penetrance of genetic variants,^17^ and population databases have already been central to confirming or refuting pathogenicity of genetic variants and validating clinical decisions.^18^ The extent to which family-based penetrance impacts variant pathogenicity through unknown risk modifiers remains unquantified, though these modifying effects are being studied in cancer cohorts.^19^ Uncertainty surrounding penetrance estimates between familial disease cohorts and unselected population cohorts could lead to the provision of inflated risk estimates and recommendations for interventions such as surgery and screening that are based on this falsely high risk.^20^ Genetic risk scores (GRS) have been increasingly used to explain some of the additional variance and in some cases treated as a proxy for family history, however recent data from FinnGen has shown that GRS and family history are independent, not interchangeable and provide complementary information.^21^

Here we use exome sequencing data and clinical records from 454,712 individuals from UK Biobank to estimate the population penetrance of pathogenic genetic variants for breast and colorectal cancer from two cancer-predisposition syndromes (HBOC and Lynch syndrome, respectively) and investigate the effect of first-degree relative (FDR) history of these cancers on these estimates.

## Methods

### Cohort

We used data from UK Biobank.^22^ Hospital Episode Statistics and cancer registry data were available for the whole cohort up to 25 June 2021, and baseline participant questionnaires. Exome sequencing data were available on 454,712 individuals (246,591 women), generated externally by Regeneron.^23^

The UK Biobank resource was approved by the UK Biobank Research Ethics Committee and all participants provided written informed consent to participate.

### Variant identification

Detailed sequencing methodology for UK Biobank samples is provided by Szustakowski et al.,^24^ exomes were captured with the IDT xGen Exome Research Panel v1.0 which targeted 39Mbp of the human genome with coverage exceeds on average 20× on 95.6% of sites. The OQFE protocol was used for mapping and variant calling to the GRCh38 reference. We included variants that had individual and variant missingness <10%, Hardy Weinberg Equilibrium p-value >10^−15^, minimum read depth of 7 for SNVs and 10 for indels, and at least one sample per site passed the allele balance threshold >15% for SNVs and 20% for indels.^25^ Variants were annotated using the Ensembl Variant Effect Predictor (VEP).^26^

### Pathogenic variant classification

Variants were considered in clinically relevant MANE select transcripts for HBOC in the *BRCA1* (ENST00000357654) and *BRCA2* (ENST00000380152) genes (hereafter collectively referred to as BRCA variants) and for Lynch syndrome in the *MLH1* (ENST00000231790), *MSH2* (ENST00000233146) and *MSH6* (ENST00000234420) mismatch repair genes. We excluded *PMS2* from our analysis due to the difficulty in variant calling caused by highly homologous sequences and pseudogene sequence exchange. We also did not include large deletions in *EPCAM*, a non-mismatch repair gene which is known to cause hypermethylation of *MSH2*. As described previously,^27^ variants in these genes were defined as pathogenic if they had been classified as pathogenic or likely pathogenic at 2∗ level or above in the ClinVar database (accessed April 2022).^28^ We also included likely protein truncating variants (PTV), which we defined as any variant that is predicted to cause a premature stop gain, a frameshift, or abolish a canonical splice site (−2 or +2 bp from exon boundary); we excluded PTVs in the last exon of each gene. Any pathogenic variants identified were confirmed visually using the Integrative Genomics Viewer (IGV)^29^ to examine the alignments of quality controlled sequencing reads. Two independent authors with extensive experience of comparing IGV data to confirmed variant calls identified likely false positive variants (for example too few reads or alternate allele reads to support the call, poor mapping to the reference sequence, incorrectly called indels as SNVs or no evidence at all for the call). All flagged variants and any discrepant judgements between reviewers were discussed and where consensus was achieved these variants were excluded. Our approach is broadly in line with that taken by Insight-ClinGen and ENIGMA for mismatch repair genes and BRCA genes respectively but by keeping to a broader set of principles rather than taking an expert-curated panel, we hope our approach can be transferred to other genes and conditions for which there is no or less expert curation.

### Cancer diagnosis and age at diagnosis

Cancer registry data for breast and colorectal cancer was collected for all UK Biobank participants with exome sequencing data. Although both BRCA and Lynch syndrome variants are linked to multiple other cancers, family history information was only available for breast and bowel cancer and so the analysis was limited to these cancer types. ICD-9 and ICD-10 codes were used to identify individuals with breast cancer (ICD-9 codes 174 (all subcodes); ICD-10 codes C50 (all subcodes)) and colorectal cancer (ICD-9 codes 153 (all subcodes), 1540 and 1541; ICD-10 codes C18, C19 and C20 (all subcodes)). The age at diagnosis was also extracted from the registry for these individuals.

### Family history calculation

UK Biobank participants were asked about 12 specific illnesses within their family as part of the enrolment process. We used the fields 20,107 (illnesses of father), 20,110 (illnesses of mother) and 20,111 (illnesses of siblings) to create a new variable of positive first-degree family history for breast and bowel cancer. Bowel cancer was specifically the term used to ask participants about their family history so has been used here but it should be noted this may have yielded different results if the question had asked about CRC. There was no information recorded on illnesses of children or second-degree family history of these conditions, nor the age at which family members were diagnosed. We did not differentiate between whether an individual had one or multiple affected family members, we simply considered a binary FH yes/no. Genetic risk scores were also generated for breast and colorectal cancer to examine their influence on family history effects (Supplementary Methods).

### Statistical testing

All data analysis and statistical testing was performed in Stata (Version 16.1). Kaplan–Meier survival analysis was carried out to assess the relationship between individuals with a pathogenic BRCA or Lynch syndrome variant and first-degree family history. BRCA analysis was restricted to women (defined here as those coded 0 (female) in field n3100 (sex) and whose genetic sex did not conflict with their reported answer to this question). A Cox linear regression model was built using mastectomy in the absence of cancer (to control for prophylactic treatment biasing outcome in BRCA carriers), breast or bowel screening, death, recruitment centre and sex (Lynch analysis only) with time set as the age of participants. We did not remove individuals who had mastectomy from our analysis but addressed this by including it in the model as described above. Removing individuals with mastectomy could introduce a bias and risks losing individuals who had a cancer-related mastectomy. Sub-group analyses were also performed on those groups stratified by positive or negative first-degree family history. The resultant model was used to predict survival functions.

Kaplan–Meier curves were generated using failure as the age when individuals were diagnosed with breast or colorectal cancer and time as the age of the participants. As we have cancer registry data going back prior to enrolment in the study, age at risk started from birth and ended at the age of participants at the final cancer registry data point we used for analysis. There was no additional censoring. Cox proportional hazard tests for equivalence of survival functions were used to interrogate inter-group differences. This test is a variation on the log-rank test and uses a COX proportional hazards model on indicator variables for each group and reports relative hazards (relative to a reference group that excludes each defining characteristic of that group) which are the exponentiated coefficients from the COX model, renormalised (https://www.stata.com/manuals13/stststest.pdf). Incidence-rate ratios were also calculated using person-time derived from the survival model, starting at birth and ending at age of participant at the last cancer registry update.

Penetrance estimates are predictions derived from the survival model.

### Meta-analysis

We conducted a meta-analysis of the effects of family history across genes been within and between the two syndromes considered. Relative risk values were generated for cancer diagnosis before age 60, for all five genes and combined analysis performed across both syndromes for all pathogenic variant carriers. We used a random effects model under the assumption that the effect of family history on pathogenic variants may vary across each gene/syndrome and assessed intergroup heterogeneity. We used cancer diagnosis before age 60 for this analysis.

### Role of funding

The current work is supported by the MRC (grant no MR/T00200X/1). The MRC had no role in the design and conduct of the study; collection, management, analysis, and interpretation of the data; preparation, review, or approval of the manuscript; and decision to submit the manuscript for publication.

## Results

### Women with a pathogenic BRCA variant and a FDR family history of breast cancer have a significantly increased risk of breast cancer compared to those with a pathogenic BRCA variant alone

We identified 230 women with a pathogenic variant in *BRCA1* (*BRCA1*+) and 611 in *BRCA2* (*BRCA2*+). Carriers were further categorised into those who had a FDR with breast cancer (FH+; n = 78 for *BRCA1* and n = 170 for *BRCA2*) and those who did not (FH-; n = 152 for *BRCA1* and n = 441 for *BRCA2*).

**Fig. 1:**
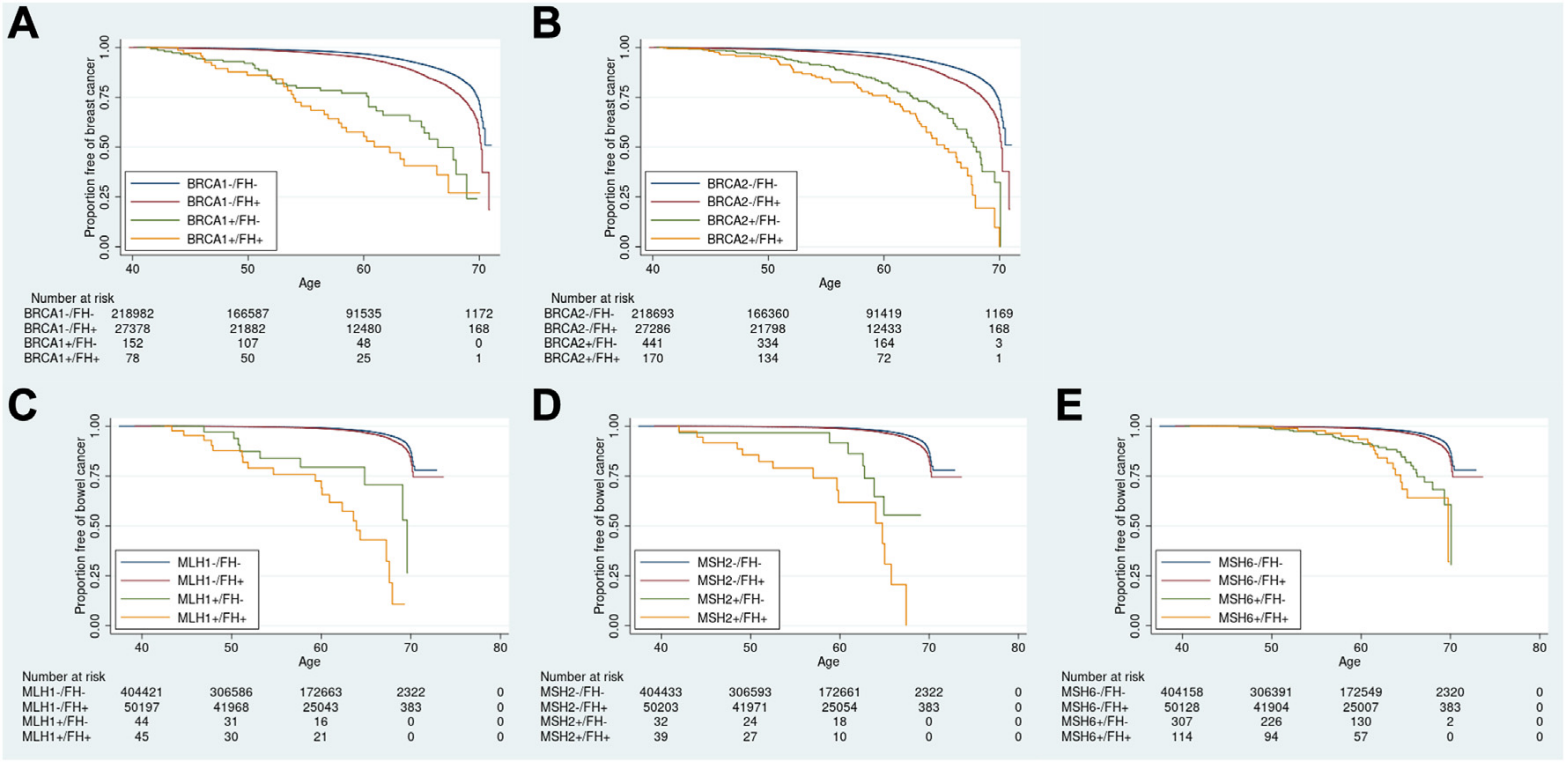
**Survival curves showing risk in UK Biobank of being diagnosed with cancer**. Participants were stratified and Kaplan–Meier survival curves plotted based on whether they had a pathogenic *BRCA1* (women only) (**A**), *BRCA2* (**B**) (women only), *MLH1* (**C**), *MSH2* (**D**) or *MSH6* (**E**) variant and/or first-degree family history of breast cancer.

**Fig. 2:**
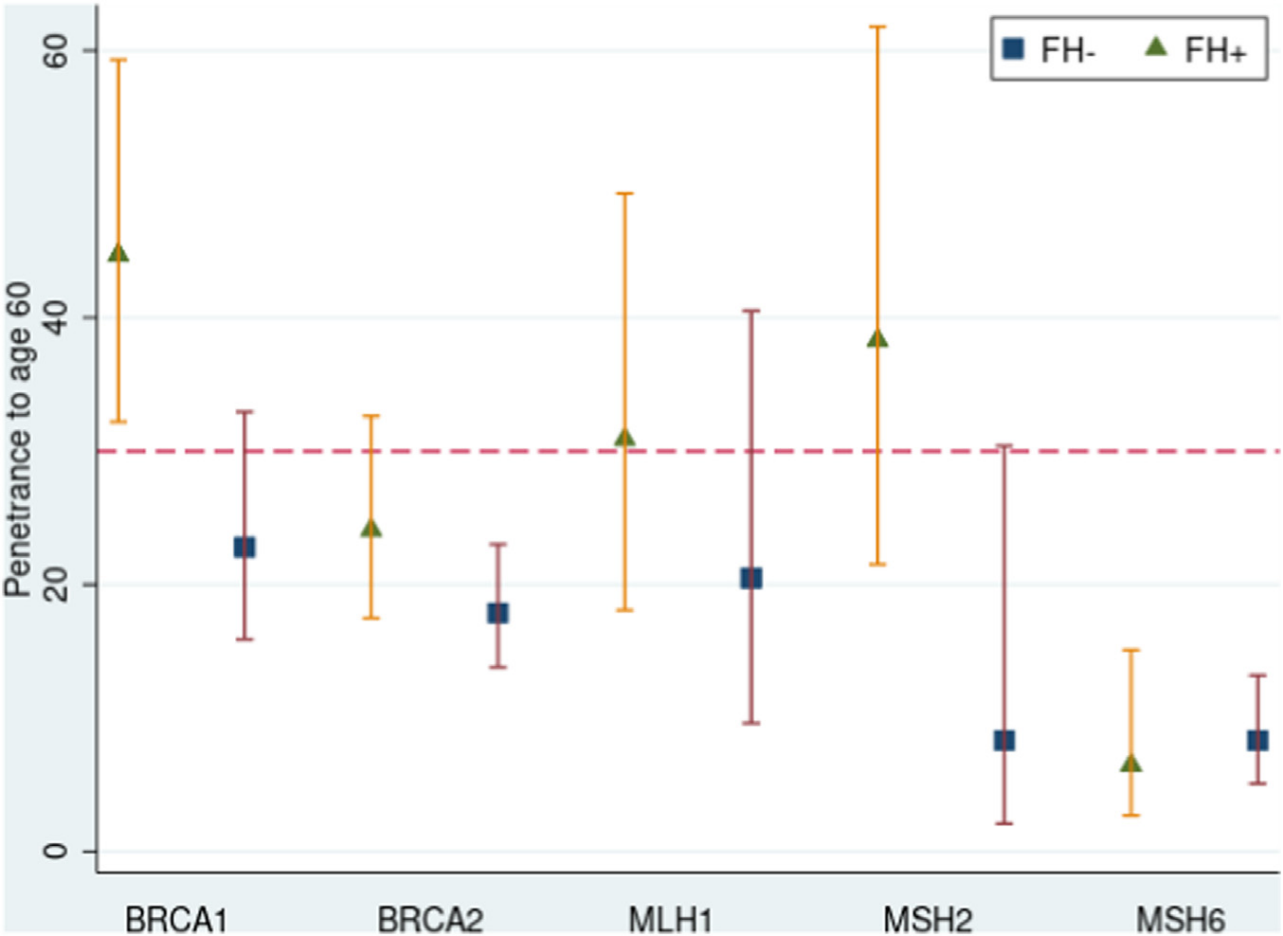
**Effect of family history on penetrance to age 60.** Penetrance to age 60 was calculated using the survival model and shown per gene, split by positive or negative family history. Error bars show the 95% confidence intervals. A red dotted line has been added at 30% to indicate the lifetime risk level (note: not risk to age 60) used by NICE to guide enhanced surveillance in women at risk of breast cancer in the UK.^30^ These guidelines consider all *BRCA1* and *BRCA2* carriers to be high risk. The latest Mallorca guidelines for Lynch syndrome also suggest enhanced surveillance for all *MLH1*, *MSH2* and *MSH6* carriers.^31^ This consists of 2 yearly colonoscopy from age 25 for *MLH1/MSH2* carriers and age 35 for *MSH6* carriers.

Kaplan–Meier curves were generated for the different groups (i.e., BRCA ± and family history +/−) and the Cox regression-based test for equality of survival curves demonstrated a significant difference between variant carriers with and without family history for both *BRCA1* (chi^2^ = 604.27, p < 0.0001, relative hazard 10.29 with family history and 7.24 without) and *BRCA2* (chi^2^ = 689.63, p < 0.0001, relative hazard 7.82 with family history and 4.66 without) (Fig. 1). The survival model predicts a significantly increased penetrance (chi^2^ = 11.7 p < 0.001) to age 60 in *BRCA1*+/FH + women (44.7% 95% CI 32.2–59.3) compared to *BRCA1*+/FH- women (22.8% 95% CI 15.9–32.0). The predicted penetrance to age 60 in *BRCA2*+/FH + women was 24.1% (95% CI 17.5–32.6) versus 17.9% (95% CI 13.8–23.0) in *BRCA2*+/FH- women, though this difference was not statistically significant (Fig. 2). Incidence-rates were also significantly higher in both BRCA1+/FH+ (rate ratio 1.5 p = 0.04, person-time FH+ 4239.6 FH- 8325.3) and BRCA2+/FH+ (rate ratio 1.7 p < 0.0001, person-time FH+ 9671.5 FH- 24732,8) women compared to those who were FH-. GRS did not explain the family history differences (Supplementary results and Supplementary Figure S1).

### Individuals with a pathogenic Lynch variant and a FDR family history of bowel cancer have an increased risk of colorectal cancer compared to those with a pathogenic Lynch variant alone

We identified 89 individuals with a pathogenic variant in *MLH1* (*MLH1*+), 71 in *MSH2* (*MSH2*+) and 421 in *MSH6* (*MSH6*+). Carriers were further categorised into those who had a FDR with bowel cancer (FH+; n = 45 for *MLH1*, n = 39 for *MHS2* and n = 114 for *MSH6*) and those who did not (FH-; n = 44 for *MLH1*, n = 32 for *MHS2* and n = 307 for *MSH6*).

Kaplan–Meier curves were generated for the different groups and the Cox regression-based test for equality of survival curves demonstrated a significant difference between variant carriers with and without family history for *MLH1* (chi^2^ = 226.7, p < 0.0001, relative hazard 35.6 with a family history and 13.0 without), *MSH2* (chi^2^ = 207.6, p < 0.0001, relative hazard 48.0 with a family history and 15.4 without) and *MSH6* (chi^2^ = 217.7 p < 0.0001, relative hazard 9.9 with a family history and 7.2 without) (Fig. 1). The survival model predicts an increased penetrance to age 60 in *MLH1*+/FH + individuals (30.9% 95% CI 18.2–49.3) compared to *MLH1*+/FH- individuals (20.5% 95% CI 9.6–40.5). For *MSH2*+ individuals, the penetrance to age 60 was 38.3% (95% CI 21.5–61.8) for FH+ and 8.3% (95% CI 2.1–30.4) for FH-; for *MSH6*+ individuals, the penetrance to age 60 was 6.5% (95% CI 2.7–15.1) for FH+ and 8.3% (95% CI 5.1–13.2) for FH- (Fig. 2), trending in the opposite direction to the other genes. None of the predicted differences in penetrance to age 60 were statistically significant in the survival model. Incidence-rate comparison showed a significant difference for *MLH1*+/FH+ (rate ratio 2.2 p = 0.03, person-time FH+ 2525.1 FH- 2447.5) but not *MSH2*+/FH+ (rate ratio 1.8 p = 0.09, person-time FH+ 2124.8 FH- 1830.8) or *MSH6*+/FH + individuals (rate ratio 1.4 p = 0.15, person-time FH+ 6609.1 FH- 17431.9) compared to FH-.

### Combined analysis across all genes showed a consistent trend for significantly elevated risk of cancer in pathogenic variant carriers with a FDR family history versus those without

**Fig. 3:**
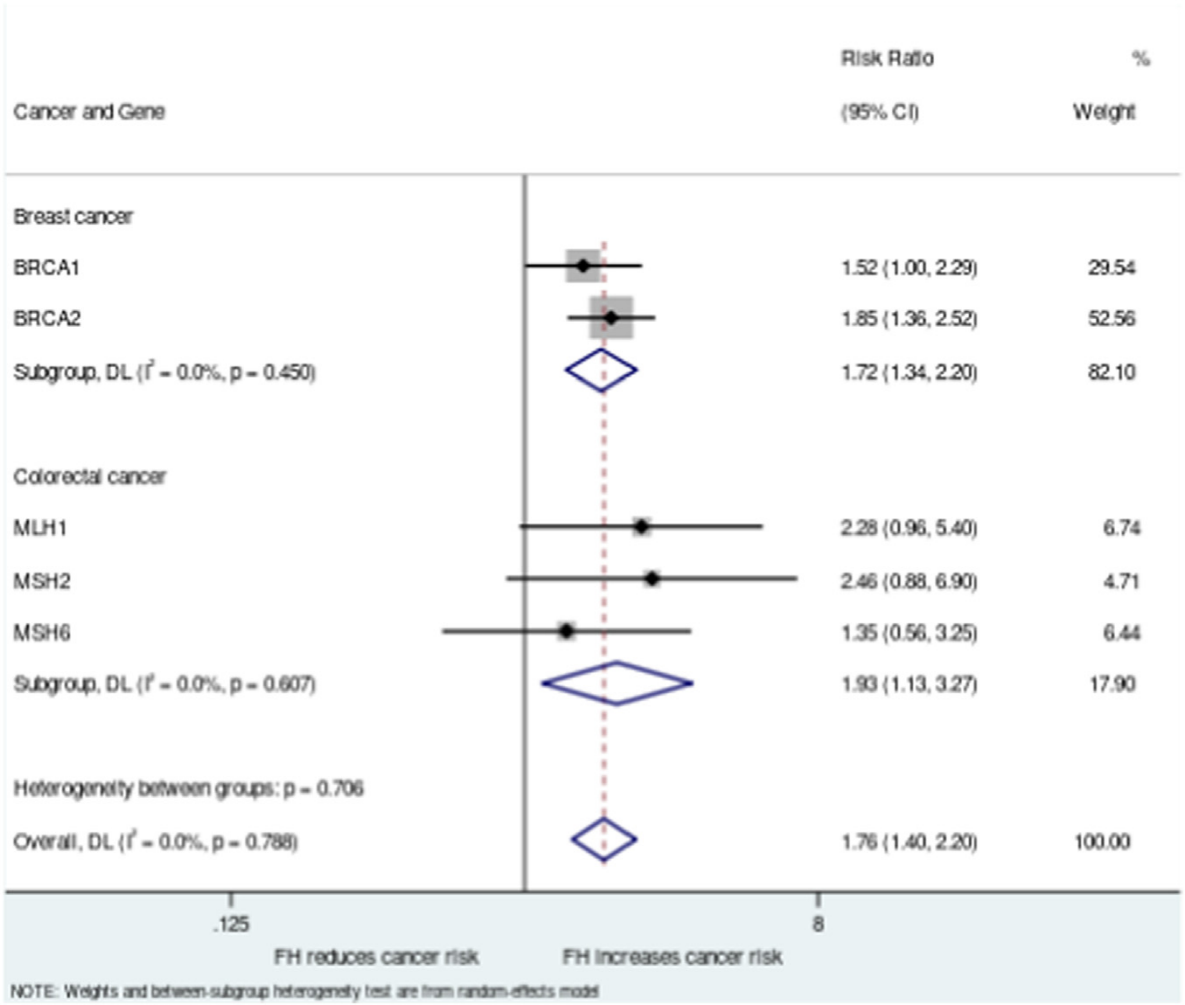
**Effect of family history on risk ratio.** The risk ratio for cancer diagnosis based on presence or absence of family history was analysed for women with *BRCA1* or *BRCA2* pathogenic variants and all individuals with *MLH1, MSH2* or *MSH6* variants. Error bars show the 95% confidence intervals. Individual gene level analysis was performed along with combined analyses per syndrome and across all genes using a random effects model.

Relative risk values were generated for all five genes and combined analysis performed across both syndromes for all pathogenic variant carriers, giving an overall increased risk of cancer in those with a family history of 1.76 (95% CI 1.40–2.20) versus those without (Fig. 3). Subgroup analyses for breast cancer (*BRCA1/BRCA2*) and colorectal cancer (*MLH1/MSH2/MSH6*) were also significant, with relative risks of family history of 1.72 (95% CI 1.34–2.20) and 1.93 (95% CI 1.13–3.27) respectively.

## Discussion

Using data from a large population cohort, we have shown that much of the risk conferred by a rare pathogenic variant associated with HBOC or Lynch syndrome is conferred by a FDR family history of disease. In UK Biobank, women with a *BRCA1*/2 variant are 1.5/1.9-times more likely to get breast cancer if they also have a first-degree family history of breast cancer, whilst individuals with a *MLH*1/*MSH*2/*MSH*6 variant are 2.3/2.5/1.4-times more likely to get colorectal cancer if they also have a first-degree family history of bowel cancer. Carriers with a family history are also more likely to develop cancer earlier versus those without. These risk increases are consistent with those previously observed in clinically ascertained cohorts^32^ but have not previously been estimated in a population cohort. This difference in penetrance in carrier individuals, if replicated in larger studies, could be sufficient to justify stratifying just individuals with a family history into high-risk groups currently eligible for specialist clinical care (e.g., NICE guidance on familial breast cancer requires ≥30% lifetime risk to be eligible for referral to a specialist genetic clinic in the UK, whilst all Lynch syndrome patients are suggested to have 2 yearly colonoscopy from age 25 for *MLH1/MSH2* carriers and age 35 for *MSH6* carriers).^30^ We analysed breast and colorectal cancer genetic risk scores and found these did not explain the difference in FH groups (Supplementary discussion). However, a combined algorithm incorporating GRS, family history, monogenic variant and other lifetime risk factors could be extremely useful in stratifying patients ascertained in a genotype-first manner, matching the approaches currently used in familial cohorts.

Our results are consistent with comparable population studies of cancer susceptibility, but to the best of our knowledge, our study is the first to investigate the effect of family history alone in a clinically unselected population across multiple syndromes. The penetrance of *BRCA1/2* mutations was previously estimated in 49,960 individuals in UK Biobank, but the analysis did not evaluate family history.^33^ Among pathogenic variant carriers for both HBOC and Lynch syndrome, the probability of disease by age 75 has been estimated to range from 13 to 76% for breast cancer and 11–80% for colon cancer respectively, based on different polygenic background, but again this analysis did not specifically analyse the effect of having a first-degree relative with the disease.^34^ Recent work in a smaller subset of UK Biobank has also shown consistent results in colorectal cancer, highlighting the added value of family history in combination with polygenic risk scores.^35^ The penetrance of HBOC amongst clinically unselected pathogenic *BRCA1/2* variant carriers was previously shown to be significantly different between those with and without a family history (83% versus 60% to age 60 for *BRCA1*, and 76% versus 33% to age 80 for *BRCA2*), but the study was limited to just three variants that are relatively common in the Ashkenazi Jewish population of Israel.^36^

Our findings are particularly important when considering reporting of secondary findings in individuals who are undergoing genomic sequencing for another indication. All five genes investigated here are included on both the ACMG Secondary Findings list^14^ and the UK 100,000 Genomes Project^13^ additional findings list due in part to their clinical actionability. This represents an ‘opportunistic screening’ scenario for these variants. Using UK Biobank, we can calculate the hypothetical number of people that would need to be assessed for pathogenic variants in these five cancer syndrome genes to prevent one case of hereditary breast or colorectal cancer, and consequently the number who could be harmed unnecessarily by follow-on interventions. There were 8635 women diagnosed with breast cancer and 2269 individuals diagnosed with colorectal cancer before the age of 60 in ∼454,000 individuals with exome sequencing data. Analysing pathogenic variants in all individuals would identify 189 BRCA + women who went on to get cancer before the age of 60. Therefore, you would need to sequence 1305 women to prevent one breast cancer diagnosis. However, for each diagnosis potentially prevented, around 3 women could be exposed to needless breast surveillance or prophylactic treatment. With current estimates suggesting around 30% of women (more than 50% in the USA) with pathogenic BRCA variants opt for prophylactic bilateral mastectomy, this could mean one needless mastectomy for every true case of breast cancer. Small sample size of Lynch positive individual and lack of specificity in the family history data (bowel cancer versus CRC) limited our CRC analysis. However, if CRC is found to be significant in larger studies, there would be similar implications for colonoscopy.

Whilst existing family-based cohorts suffer from an ascertainment bias that is likely to overinflate penetrance estimates, the older cohort in the UK Biobank is likely to be confounded by survival bias,^37^ i.e., individuals with the most severe early-onset disease will not appear as they would have died prior to recruitment. Despite taking a conservative approach to pathogenic variant classification, this bias will have the effect of removing very highly penetrant variants from the cohort, which is likely to deflate penetrance estimates. To estimate the size of this problem in UK Biobank, we investigated the frequency of pathogenic BRCA variants in men versus women. A large over-representation in men would suggest we are missing a number of early breast cancer cases. Overall, pathogenic BRCA variants are present in 0.34% (840/246,591) women and 0.38% (800/208,121) men in our cohort, whilst this difference wasn't statistically significant, it suggests the possibility of around an 11% depletion of women with pathogenic BRCA variants and early onset terminal breast or ovarian cancer, which is consistent with other population cohorts.

The true penetrance for an unselected population is likely to be somewhere between the figures generated from each context. Limiting our analyses to just breast and colorectal cancer (to match the available family history information) will also act to underestimate the true penetrance of these variants in causing any cancer. The UK Biobank is also not a representative population cohort, due to recognised recruitment biases,^38^ and so these estimates are likely to represent a lower bound.

Additionally, a full family history was not recorded for participants in UK Biobank, so we relied upon self-reported illness in FDR as a proxy for family history and were only able to include breast and bowel cancers. Given the age of the UK Biobank cohort (recruited from 40 to 69 years old), these recollections could be biased towards more aggressive or early-onset disease, particularly in parents. The lack of second-degree family history information or that concerning children will also mean our cohort differences will be underestimated due to the presence of individuals with a family history in the FH negative group. We were unable to consider age of diagnosis of family members as this data was not present, nor were we powered to consider number of affected family members due to the small numbers in some sub-groups. Larger studies will be able to assess the impact of this on refining the penetrance estimates.

Survival analysis, whilst a common and appropriate method for analysing this type of data is restricted by a number of assumptions. The Cox model assumes proportional hazards over time and linearity, which may not hold true for inherited cancer cohorts and may impact the estimates derived. The varying age of participants in UKBB also means that censoring on the right hand side of the survival curves results in incomplete data. This is partly why we decided to use age 60 as a penetrance cut-off due to more complete data at this timepoint.

Finally, due to the rarity of individual pathogenic variants, we were limited by the size of the existing cohort. The small numbers of MLH1 and MSH2 variant carriers in particular may limit the actionabaility of our findings when considering guideline revisions. We have attempted to mitigate these issues slightly by including data from all eligible participants in UK Biobank (n = 454,712), regardless of ethnicity or consanguinity. We are underpowered to detect differences in penetrance between individual variants, which will also be important for risk discussions with patients. Future research with larger cohorts is needed further improve risk prediction and investigate modifiers.

The findings of this study suggest that any universal policy of returning pathogenic cancer predisposing genetic variants found incidentally or through direct-to-consumer genetic testing of asymptomatic individuals should consider family history and other factors when counselling patients on the risks and benefits of follow-up care. It will be very difficult to counsel individuals as to their particular risk profile without further pedigree construction or investigations. If penetrance estimates from affected families are used, there is a danger of over-management of asymptomatic individuals with no family history of disease. These “patients-in-waiting” may be exposed to unnecessary surveillance or more invasive prophylactic procedures.^6^^,^^7^ Follow-up data gathered from such initiatives as the UK 100,000 Genomes Project^13^ and the American College of Medical Genetics and Genomics Recommendations on Reporting of Secondary Findings^14^ will be critical to decipher the exact risk profile of unselected variant carriers. It is imperative that individuals who receive genotype information indicating a predisposition to cancer are appropriately counselled as to their individual risk profile in the context of their family history of disease. For those ascertained outside of the standard clinical pathway, this will help to avoid patients at risk levels not far above background making injudicious decisions about prophylactic options.

It has long been known, though is not widely appreciated, that penetrance estimates for pathogenic variants causing hereditary subtypes of common diseases are likely to be significantly inflated due to ascertainment bias.^12^ The use of selection criteria for genetic testing based on multiple affected family members^3^^,^^31^ will necessarily bias the findings towards those families in whom the variants have a high penetrance. We have shown that, even in a clinically unselected population, having an affected first-degree relative may increase the penetrance of pathogenic variants for breast and colorectal cancer in two hereditary cancer syndromes. Systematic testing either of all patients with breast or colorectal cancer or in truly unselected populations is likely to yield more conservative estimates.

## Contribution

ARW, JT & KSR–data curation, methodology, software, writing–review & editing.

JWH–data curation, formal analysis, investigation, validation, writing–review & editing.

CFW & MNW–Conceptualization, Data curation, funding acquisition, investigation, methodology, resources, software, validation, writing–original draft & writing–review & editing.

LJ–Conceptualization, Data curation, formal analysis, investigation, methodology, project administration, resources, validation, visualisation, writing–original draft & writing–review & editing.

LJ, CFW & MNW accessed and verified the data.

## Data sharing statement

All data used in this study can be accessed via application to UK Biobank and approval via the data access committee. The authors are not permitted to directly share this data. The code used to perform GRS is available at https://github.com/hdg204/Rdna-nexus. Variants classified as pathogenic and used in this study are defined in the methods and can be replicated, however a list is available on request.

## Declaration of interests

The authors declare no competing interests.

## Figures and Tables

**Supplementary Figure S1:**