# Supplementary material for: Influence of family history on penetrance of hereditary cancers in a population setting
Source: eClinicalMedicine. 2023 Sep 14;64:102159. doi: 10.1016/j.eclinm.2023.102159 (PMC10626157; doi:10.1016/j.eclinm.2023.102159)
Supplement: Supplementary Material [file mmc1.docx]

# Supplementary Material

## Supplementary Methods

### **Generation of genetic risk scores (GRS)**

A genetic risk score of 306 SNPs for breast cancer was calculated using the BRIDGES GRS variation as described in a recent paper comparing different PRS approaches, using weights from Supplementary Table S4[1].

A genetic risk score of 207 SNPs for colorectal cancer was calculated using a recent meta-analysis of 100,204 cases and 154,587 controls from mixed European and east Asian ancestry[2], using weights in Supplementary Table 4.

GRSs were calculated by multiplying the beta for each SNP by the individual’s genotype for each risk allele, using imputed genotype data from the UK Biobank. The code used to perform this calculation is available at [https://github.com/hdg204/Rdna-nexus](https://eur03.safelinks.protection.outlook.com/?url=https%3A%2F%2Fgithub.com%2Fhdg204%2FRdna-nexus&data=05%7C01%7CL.Jackson2%40exeter.ac.uk%7C9d10d1a65b3e4af9119708db72573ccd%7C912a5d77fb984eeeaf321334d8f04a53%7C0%7C0%7C638229491218506085%7CUnknown%7CTWFpbGZsb3d8eyJWIjoiMC4wLjAwMDAiLCJQIjoiV2luMzIiLCJBTiI6Ik1haWwiLCJXVCI6Mn0%3D%7C3000%7C%7C%7C&sdata=3ksUBllymDke%2BfWwXg7n0yuJRB8qEi4btjOL3kvd8Hs%3D&reserved=0).

**Supplementary Results**

We constructed GRSs for breast and colorectal cancer. The breast cancer GRS had a ROC AUC of 0.626 and the colorectal GRS had a ROC AUC of 0.627. We stratified pathogenic variant carriers by GRS(low/high) and family history and examine the effects on cancer diagnosis (Supplementary Figure 1). In contrast to the variant and family history survival curves, risk did not stratify consistently by GRS.

**Supplementary discussion**

It could be suggested that our findings on family history increasing penetrance of pathogenic variants could be down to GRS differences in these groups. We have shown that in our data, the risk does not overlap with GRS, in fact across some of the genes we consider, GRS-low individuals with a family history are at higher risk than GRS-high individuals. This is in accordance with recent data from FINNGEN confirming that GRS and family history data are independent and complimentary data sources and should not be used interchangeably[3].

**Supplementary Figure Legend**

Supplementary Figure 1. **Survival curves showing risk in UK Biobank of being diagnosed with cancer.**

Participants were stratified and Kaplan-Meier survival curves plotted based on whether they had a pathogenic *BRCA1* (women only) (**A**), *BRCA2* (**B**) (women only), *MLH1* (**C**), *MSH2* (**D**) or *MSH6* (**E**) variant, a high or low GRS for breast cancer (**A** & **B**) or colorectal cancer (**C**, **D** & **E**) and/or first-degree family history (FH) of breast cancer.

Supplementary References

1. Mavaddat, N., et al., *Incorporating Alternative Polygenic Risk Scores into the BOADICEA Breast Cancer Risk Prediction Model.* Cancer Epidemiol Biomarkers Prev, 2023. **32**(3): p. 422-427.

2. Fernandez-Rozadilla, C., et al., *Deciphering colorectal cancer genetics through multi-omic analysis of 100,204 cases and 154,587 controls of European and east Asian ancestries.* Nat Genet, 2023. **55**(1): p. 89-99.

3. Mars, N., et al., *Systematic comparison of family history and polygenic risk across 24 common diseases.* Am J Hum Genet, 2022. **109**(12): p. 2152-2162.
